# Next-Generation Therapies for Multiple Myeloma

**DOI:** 10.1146/annurev-cancerbio-061421-014236

**Published:** 2024-01-11

**Authors:** Erin W. Meermeier, P. Leif Bergsagel, Marta Chesi

**Affiliations:** 1 Department of Immunology, Mayo Clinic, Scottsdale, Arizona, USA; 2 Department of Medicine, Mayo Clinic, Scottsdale, Arizona, USA

**Keywords:** multiple myeloma, T cell engagers, bispecific antibody, chimeric antigen receptor, CAR T cell, superenhancers, EP300

## Abstract

Recent therapeutic advances have significantly improved the outcome for patients with multiple myeloma (MM). The backbone of successful standard therapy is the combination of Ikaros degraders, glucocorticoids, and proteasome inhibitors that interfere with the integrity of myeloma-specific superenhancers by directly or indirectly targeting enhancer-bound transcription factors and coactivators that control expression of MM dependency genes. T cell engagers and chimeric antigen receptor T cells redirect patients’ own T cells onto defined tumor antigens to kill MM cells. They have induced complete remissions even in end-stage patients. Unfortunately, responses to both conventional therapy and immunotherapy are not durable, and tumor heterogeneity, antigen loss, and lack of T cell fitness lead to therapy resistance and relapse. Novel approaches are under development to target myeloma-specific vulnerabilities, as is the design of multimodality immunological approaches, including and beyond T cells, that simultaneously recognize multiple epitopes to prevent antigen escape and tumor relapse.

## 1. INTRODUCTION

**Figure 1:**
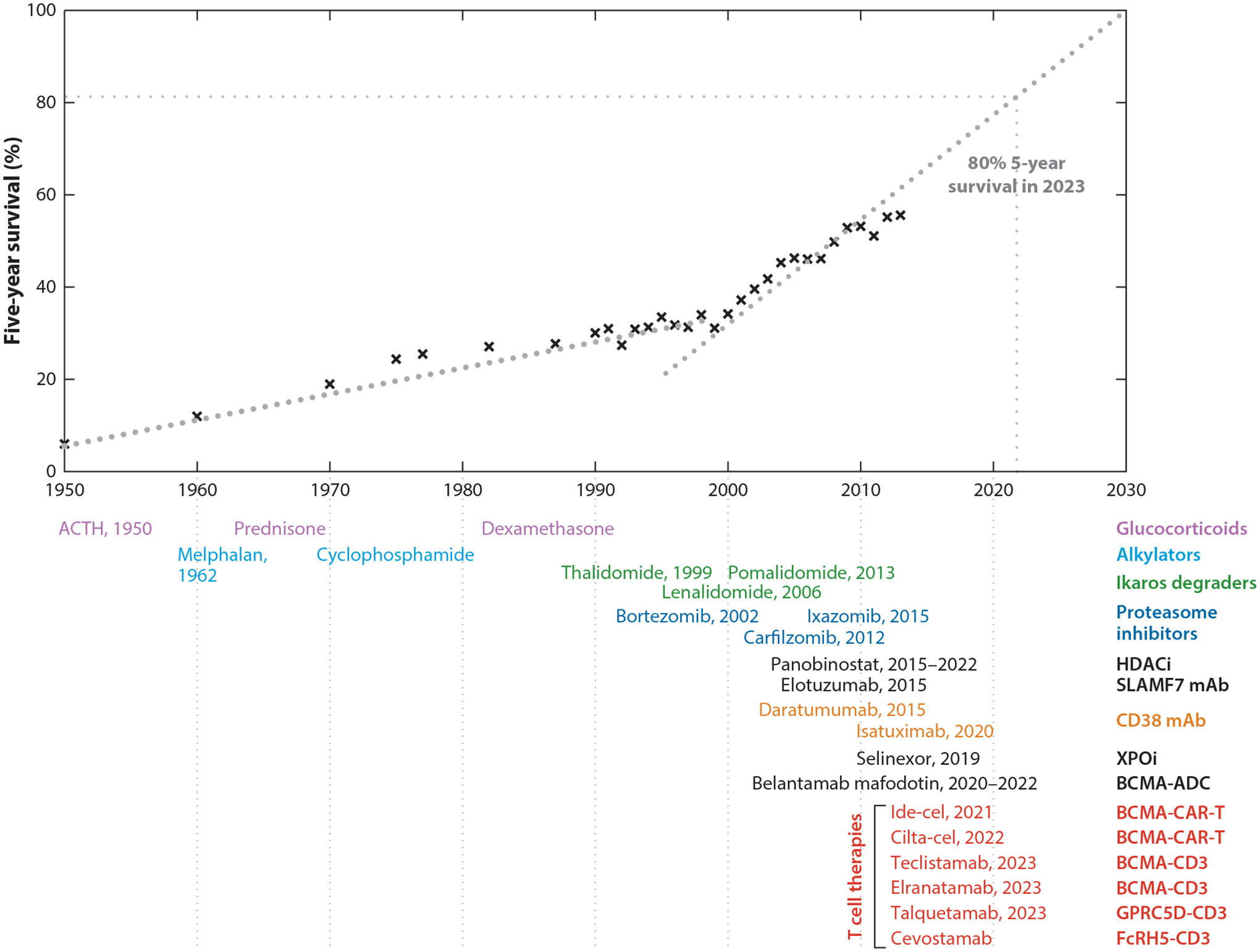
Timeline of introduction and FDA approval of drugs for the treatment of MM. Drugs are colored on the basis of the six main classes to which they belong. Drugs colored in black have relatively unique mechanisms of action and more limited use. For two of these (panobinostat and belantamab mafodotin), FDA approval was withdrawn in 2022. Abbreviations: ACTH, adrenocorticotropic hormone; ADC, antibody drug conjugate; BCMA, B cell maturation antigen; CAR, chimeric antigen receptor; cilta-cel, ciltacabtagene autoleucel; ide-cel, idecabtagene vicleucel; FcRH5, Fc receptor homolog 5; FDA, US Food and Drug Administration; GPRC5D, G protein–coupled receptor, class C group 5 member D; HDACi, histone deacetylase inhibitor; mAb, monoclonal antibody; MM, multiple myeloma.

Multiple myeloma (MM) is characterized by multiple bone marrow–localized tumors composed of malignant plasma cells that lead to hypercalcemia, renal insufficiency, anemia, and lytic bone disease. The incidence and death rates per 100,000 (2015–2019) are 7 and 3.1 among all Americans and 14.3 and 5.9 among African Americans (Fuchs 2023). MM is almost always preceded for many years by an often-unrecognized premalignant condition, monoclonal gammopathy of undetermined significance (MGUS), characterized by a stable serum monoclonal immunoglobulin (Ig) and a low level (<10%) of bone marrow plasmacytosis that progresses to MM at a rate of approximately 1% per year. For many years the only effective treatments were melphalan and prednisone, first introduced in the early 1960s (Bergsagel et al. 1962). Remarkably, these treatments remain a part of the standard frontline therapy of patients today, in which an administration of one single high dose (200 mg/m^2^) of melphalan is followed by autologous peripheral blood hematopoietic stem cell transplantation (Attal et al. 1996) and dexamethasone largely supplants prednisone as the glucocorticoid of choice. The approval of Ikaros degraders (thalidomide, lenalidomide, and pomalidomide, in order of potency), starting in 1999 (Lacy et al. 2009, Singhal et al. 1999, Weber et al. 2007), and proteasome inhibitors (bortezomib, carfilzomib, and ixazomib), starting in 2002 (Moreau et al. 2016, Richardson et al. 2003, Stewart et al. 2015), revolutionized the treatment of MM and was associated with quadrupling the annual rate of improvement in 5-year survival from 0.5% to 2% (Figure 1). The next big advance came in 2015 with anti-CD38 monoclonal antibodies (mAbs) (daratumumab and isatuximab) (Attal et al. 2019, Lokhorst et al. 2015). In 2023, the therapy for newly diagnosed MM with the longest progression free survival (PFS) is an induction with a combination of an anti-CD38 mAb, a proteasome inhibitor, lenalidomide, and dexamethasone (Voorhees et al. 2020), followed by consolidation with high-dose melphalan and autologous stem cell transplantation, and then maintenance lenalidomide until progression. B cell maturation antigen (BCMA)–chimeric antigen receptor (CAR) T cell therapies [ciltacabtagene autoleucel (cilta-cel) and idecabtagene vicleucel (ide-cel)] (Berdeja et al. 2021, Munshi et al. 2021), first approved in 2021, have shown remarkable activity in very advanced refractory MM, and ide-cel is quickly becoming the preferred therapy for first relapse (San-Miguel et al. 2023). Finally, several bispecific antibodies (BsAbs) (teclistamab, elranatamab, and talquetamab) were approved in 2023 (Chari et al. 2022a, Lesokhin et al. 2023, Moreau et al. 2022). The histone deacetylase inhibitor panobinostat (San-Miguel et al. 2014), the mAb elotuzumab (Lonial et al. 2015), the inhibitor of exportin-1 selinexor (Chari et al. 2019), and the antibody drug conjugate belantamab mafodotin (Lonial et al. 2020) are also approved for the treatment of MM, but their clinical use is limited by lack of activity or excessive toxicity.

## 2. FIVE MOLECULAR SUBTYPES OF MULTIPLE MYELOMA

MM is the malignant counterpart of terminally differentiated plasma cells that home to the bone marrow after having successfully completed the B cell receptor affinity maturation process and class switch recombination in the germinal center. It can be broadly divided into five genetic subgroups: those with one of three recurrent immunoglobulin gene translocations (45%), those with hyperdiploidy (45%), and those with neither (10%). The three translocation groups are (*a*) those that dysregulate a cyclin D gene, predominantly t(11;14) (*CCND1*) but also t(12;14) (*CCND2*) and t(6;14) (*CCND3*), present in 20% of patients; (*b*) those that dysregulate a MAF family transcription factor, predominantly t(14;16) (*MAF*) but also t(14;20) (*MAFB*) and t(8;14) (*MAFA*), present in 8% of patients; and (*c*) t(4;14), which dysregulates *NSD2* and *FGFR3*, present in 15% of patients. The breakpoints occur mostly in the switch regions of the heavy-chain locus, suggesting they occur as errors during the process of isotype class switch recombination in the germinal center. The patients with hyperdiploidy lack these recurrent IgH translocations and are characterized by trisomies of chromosomes 3, 5, 7, 9, 11, 15, 19, and 21 (Kuehl & Bergsagel 2012). A unifying feature of all the genetic subtypes of MM is the ectopic expression of a cyclin D gene because of chromosome translocation, direct transactivation of *CCND2* by a *MAF* family gene, or indirect transactivation of a low level of *CCND1* and/or *CCND2* by unknown mechanisms (Bergsagel et al. 2005). These genetic subtypes are surmised to result from the primary genetic event present in every tumor cell, starting from MGUS.

Progression from MGUS to smoldering multiple myeloma (SMM) to MM involves the step-wise accumulation of secondary genetic events. The best characterized are rearrangements of the *MYC* locus that are not identified in MGUS but are present in half of MM cases (Affer et al.2014). One-quarter of patients with MM have a variety of mutations that activate the alternative NFκB pathway, which transactivates *MYC* (Keats et al. 2007). Mutations of the RAS/mTORC pathway (*NRAS*, *KRAS*, *FGFR3*, *BRAF*, *NF1*, *PTPN11*) are present in half of patients with MM. Biallelic inactivations of *TP53*,*RB1*,and *CDKN2C* are infrequent at diagnosis but more frequent at relapse, where they are associated with extremely aggressive disease and poor outcome (Maura et al.2019).

Unlike for chronic myeloid leukemia, in which the t(9;22) Philadelphia chromosome creates a BCR–ABL1 fusion protein that can be effectively targeted by imatinib, leading to complete and durable remissions (Druker et al. 2006), no drugs are currently approved to target the primary genetic events in MM. However, an ongoing phase I trial is evaluating the efficacy of a first-in-class NSD2 histone H3K36 methyltransferase inhibitor (Zhang & Zha 2023) (https://www.clinicaltrials.gov/ identifier NCT05651932). Therapies targeting tumor-specific oncogenic mutations in MM have been unsuccessful, likely because they have been directed to progression events (i.e., MAPK pathway, AKT), which tend to be subclonal due to tumor heterogeneity, and in general are not effective due to the rapid emergence of alternative subclones. This is illustrated by elegant molecular profiling of a patient with MM with t(4;14) expressing a mutated, constitutively active *FGFR3* in which treatment with the FGFR3 inhibitor erdafitinib led to complete eradication of the *FGFR3* mutated clone and its replacement by a preexisting clone with del17p, without measurable clinical benefit (Croucher et al.2023). A similar scenario occurred after treatment with dabrafenib and trametinib (Le Calvez et al. 2020). Overall, there are no agents currently approved to target tumor-specific oncogenic mutations in MM.

## 3. THE BACKBONE DRUGS OF CURRENT MULTIPLE MYELOMA THERAPY SHARE SUPERENHANCERS AS ONE OF THEIR MANY TARGETS

**Figure 2:**
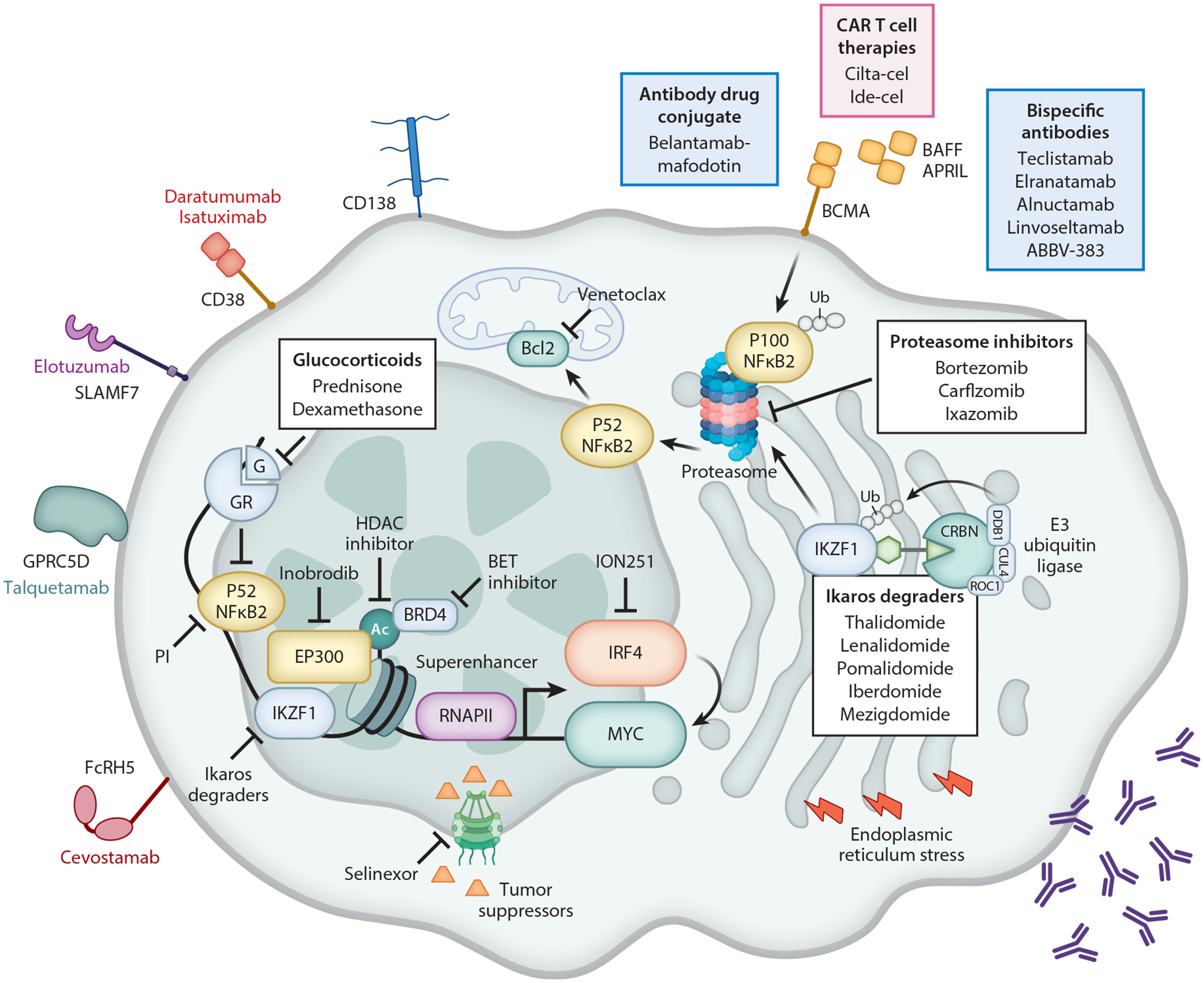
Myeloma cell biology and therapeutic clinical targets. Myeloma is the malignant counterpart of a terminally differentiated bone marrow plasma cell that secretes a large amount of monoclonal antibody. This puts the endoplasmic reticulum under continuous stress, further aggravated by PIs, creating a unique vulnerability. PIs also prevent NFκB activation by inhibiting processing of the inactive NFκB2 P100 to its active form, P52. NFκB is a key survival factor for MM cells, at least in part by promoting the transcription of prosurvival protein BCLX_L_ and BCL2, the latter being a direct target of venetoclax. NFκB, with IKZF1 and other transcription factors, also plays a central role in maintaining the activity of myeloma superenhancers that drive the expression of *MYC* and *IRF4*, key MM dependencies. Transcription factors bind to superenhancers and recruit EP300 to deposit the H3K27 acetyl marks that, once read by BRD4, allow the recruitment of RNAPII and transcription elongation. Several classes of drugs impair superenhancer function: PIs and glucocorticoids directly or indirectly target NFκB, while Ikaros degraders and ION251 induce degradation of IRF4 and IKZF1, respectively; BET, HDAC, and EP300 inhibitors target these coactivators. NFκB expression is dependent on the binding of BAFF and APRIL to their receptor BCMA, although constitutive activation of NFκB occurs in 20% of patients with MM. Because of its restricted expression in plasma cells, BCMA is an ideal target for antibody drug conjugate, bispecific antibodies, and CAR T cells. Other cell surface receptor targets in MM are CD38 and SLAMF7, which are targeted by monoclonal antibodies, and GPRC5D and FcRH5, which are targeted by bispecific antibodies. Selinexor inhibits the nuclear export of tumor suppressor genes. Abbreviations: BCMA, B cell maturation antigen; CAR, chimeric antigen receptor; FcRH5, Fc receptor homolog 5; GPRC5D, G protein–coupled receptor, class C group 5 member D; GR, glucocorticoid receptor; HDAC, histone deacetylase; MM, multiple myeloma; PI, proteasome inhibitor; RNAPII, RNA polymerase II. Figure adapted from images created with BioRender.com.

Like normal plasma cells, MM cells usually secrete a large amount of monoclonal immunoglobin, which creates a high demand for nutrients used by protein anabolism and a unique vulnerability associated with perturbations of the unfolded protein response and endoplasmic reticulum homeostasis. This activity explains at least in part the clinical success of proteasome inhibitors (Obeng et al. 2006) (Figure 2).

Ikaros degraders,whose clinical development has been empiric,are now known to bind CRBN, which is part of an E3–ubiquitin ligase complex, causing the recruitment of the transcription factors IKZF1 and IKZF3, their ubiquitination, and subsequent proteasomal degradation (Lu et al. 2014). Since the mechanism of action was discovered, even more potent IKZF1/IKZF3 degraders have been developed: cemsidomide and the CRBN E3 ligase modulator drugs iberdomide and mezigdomide. They are currently under clinical development, displaying slightly different target specificities and toxicities (Fuchs 2023).

*IKZF1* and *IKZF3* are critical dependencies for MM survival by regulating superenhancer function to maintain *MYC* and *IRF4* expression (de Matos Simoes et al. 2023, Neri et al. 2024). *MYC* dysregulation is involved in the progression of MGUS to MM, and *IRF4* is a transcription factor that is a critical dependency for MM survival (Hurt et al. 2004). Glucocorticoids transrepress NFκB-induced transcription by tethering to the transcription machinery orchestrated by CBP/EP300 at NFκB DNA binding sites (that partially overlap with glucocorticoid response elements) (Uhlenhaut et al. 2013), as do proteasome inhibitors by inhibiting the proteasome-mediated activation of NFκB (Palombella et al. 1998) (Figure 2). Not surprisingly, in a randomized clinical trial comparing glucocorticoids to proteasome inhibitors, patients with constitutive NFκB activation due to *TRAF3* inactivation had the lowest response to glucocorticoids, suggesting that excessive NFκB could not be effectively competed by glucocorticoids, and they also had the highest response to proteasome inhibitors, suggesting increased dependency on NFκB (Keats et al. 2007, Richardson et al. 2005). This finding was confirmed in a randomized clinical trial isolating the effect of ixazomib, in which patients with NFκB-activating mutations benefitted from the addition of ixazomib, whereas patients without did not (Dash et al. 2020). In contrast to mutations of the CRBN pathway that are selected upon repeated exposure to Ikaros degraders, it is puzzling that mutations of the proteasome and glucocorticoid receptors are rare. The retained efficacy of dexamethasone in multiple lines of therapy may be in part due to its ability to inhibit NFκB-driven inflammation in the tumor microenvironment, known to contribute to MM survival and disease progression.

Overall, these effective therapies for MM target the plasma cell–specific phenotype and vulnerabilities more than specific oncogenic mutations. It is notable that the three drugs that constitute the backbone of anti-MM therapy (proteasome inhibitors, Ikaros degraders, and glucocorticoids) (Durie et al. 2017) all effectively target MM superenhancers, which are essential in maintaining plasma cell identity (through *IRF4* expression), primary oncogenic events (immunoglobin translocations), and progression events (*MYC* dysregulation) (Figure 2). This may explain why patients with MM are treated similarly regardless of their genetic subgroup. An exception is the selective activity of the BCL2 inhibitor venetoclax in t(11;14) MM (Kumar et al.2020) for reasons not completely understood but attributed to t(11;14) MM having a more lymphoplasmacytic, B cell–like phenotype (Fonseca et al. 2002).

## 4. NEW SMALL MOLECULES WITH NOVEL MECHANISMS OF ACTION

Novel therapeutics are being developed to target superenhancers by inhibiting EP300-/CBP-driven H3K27 acetylation, which marks superenhancer active sites, or by competing for BRD4 binding to these acetylated marks (Loven et al.2013) (Figure 2). Although BRD4 inhibitor clinical development has suffered from excessive toxicities and lack of a therapeutic window (Amorim et al. 2016), the EP300 inhibitor inobrodib has shown promise in an early phase I trial and has now been explored in combination with pomalidomide on the basis of strong preclinical activity of the combination pomalidomide and EP300 inhibition in in vivo models (Nicosia et al. 2022, Welsh et al. 2024).

The molecular glue class of drugs functions by converting a protein into a target for an E3 ligase and includes the IKZF1/IKZF3 degraders described above. Another novel class of drugs under development similarly utilizes the cellular machinery to destroy unwanted proteins. Unlike the monofunctional molecular glues, proteolysis target chimeras (PROTACs) are hetero-bifunctional molecules that recruit target proteins to ubiquitin ligase complexes to induce their degradation (Garber 2022). In addition to IKZF1/IKZF3, such degraders have already demonstrated efficacy in targeting EP300 (Vannam et al. 2021) and CDK9, an important regulator of *MYC* and *MCL1* transcription (Olson et al. 2018). Excitingly, any unwanted protein can be degraded if a high-affinity binding site is available, offering an alternative approach to target inhibition.

## 5. IMMUNO-ONCOLOGY

A landmark 2019 multi-institution study showed that patients with penta-refractory MM (defined as refractory to an anti-CD38 mAb, two proteasome inhibitors, and two Ikaros degraders) survived a median of only 5.6 months (Gandhi et al. 2019). This sobering statistic reflected the limited therapeutic options for these patients only a few years ago and the need for new treatments to improve patient survival. The recent boon of research and approvals of additional immunotherapy, including two types of CAR T cells, to the arsenal of antimyeloma therapy have revolutionized highly efficacious therapeutic options for these patients.

**Figure 3:**
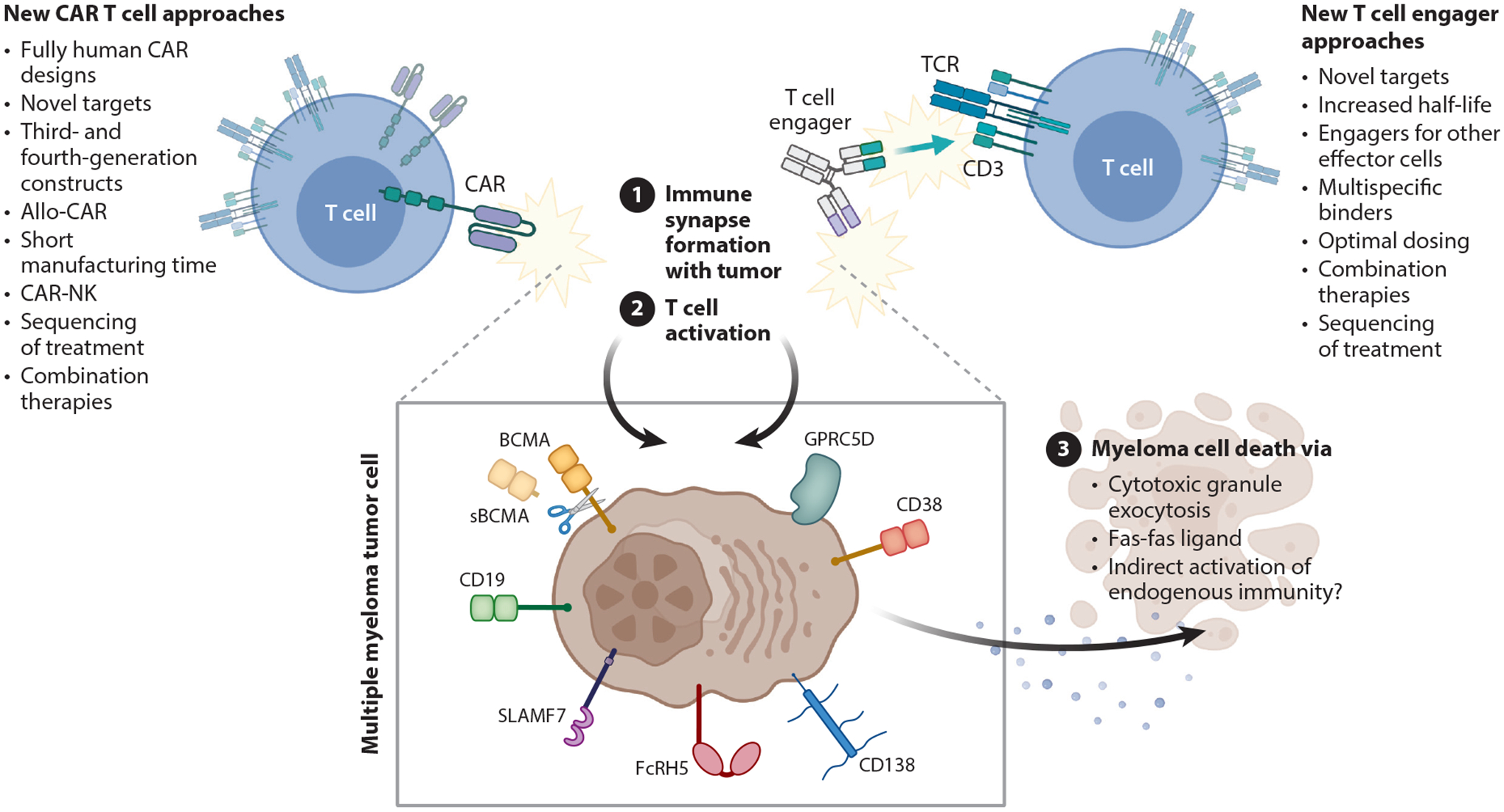
Mechanisms of action and new approaches for CAR T cell and T cell engager therapies to treat MM. Current cell therapy for MM involves engineering autologous T cells to express a CAR that recognizes specific antigens on the surface of tumor cells (depicted in the *box*), resulting in (①) immunological synapse formation, (②) T cell activation via intracellular CAR signaling domains, and (③) tumor cell killing. All CAR T cells approved to treat MM target BCMA. Numerous strategies are being evaluated to improve the clinical efficacy of CAR T cell therapy for MM (*left*). Current T cell engagers, also called bispecific antibodies, for treating MM utilize antibody-like molecules designed to simultaneously bind a tumor antigen (depicted in the *box*) and the T cell surface protein CD3, part of the common T cell receptor complex. The physical binding of CD3 on T cells leads to immunological synapse formation, T cell activation, and tumor cell killing. Numerous strategies are being evaluated to improve the clinical efficacy of T cell engager therapy for MM (*right*). Abbreviations: allo, allogeneic; BCMA, B cell maturation antigen; CAR, chimeric surface receptor; FcRH5, Fc receptor homolog 5; GPRC5D, G protein–coupled receptor, class C group 5 member D; MM, multiple myeloma; NK, natural killer cell; TCR, T cell receptor; sBCMA, soluble BCMA. Figure adapted from images created with BioRender.com.

Common current targets for immunotherapy include BCMA, CD38, SLAMF7, CD19, CD138, G protein–coupled receptor, class C group 5 member D (GPRC5D), and Fc receptor homolog 5 (FcRH5) (Figures 2 and 3). Most agents in development, and the two approved CAR T cell therapies, target BCMA and the APRIL receptor or BAFF receptor expressed on late memory B cells and plasma cells, which through NFκB activation provides survival signals for plasma cells. BCMA is cleaved from the cell surface by a gamma secretase, which we postulate serves to protect the niche of a normal plasma cell by depriving encroaching plasma cells of required survival factors but also leads to elevated levels of soluble BCMA that can interfere with BCMA-targeted therapies. CD38 and SLAMF7 are expressed on plasma cells and other immune cells and are targeted by the clinically approved mAbs daratumumab, isatuximab, and elotuzumab. CD19 is expressed on a small subset of MM cells but is an attractive target because it may be associated with self-renewal properties and drug-resistant MM subsets. CD138 is expressed at a remarkably high level by plasma cells and has been associated with tumor growth. GPRC5D is an orphan receptor expressed on malignant plasma cells. FcRH5 is expressed in B cell and plasma cell malignancies.

### 5.1. CAR T Cell Therapy

CAR T cells are autologous or allogeneic T cells engineered to express a chimeric surface receptor that recognizes specific antigens on the surface of tumor cells, resulting in T cell activation, immunological synapse formation, and target cell apoptosis (Figure 3). CARs are designed with an extracellular tumor antigen binding domain in the format of a mAb and thus bypass the normal T cell restriction to MHC-mediated antigen presentation. The intracellular domain of the CAR can contain numerous signaling modalities but always includes the CD3ζ portion of the T cell receptor for T cell activation. Other common signaling domains include those for T cell costimulation, most typically CD28 and/or 4–1BB.

#### 5.1.1. Current CAR T cell therapies.

Currently, two CAR T cell products, both of which target the antigen BCMA, are approved by the US Food and Drug Administration (FDA) for the treatment of MM. Ide-cel (bb2121) is a second-generation anti-BCMA CAR T cell product with a 4–1BB costimulatory domain. Ide-cel was approved for the treatment of relapsed/refractory MM (RRMM) based on the data of the crucial KarMMa phase 2 trial (Munshi et al. 2021). The median PFS (mPFS) for those receiving the target dose was 12.1 months and median overall survival was 24.8 months. While cytokine release syndrome (CRS) was the most common safety concern, only 6% of patients had CRS of grade 3 or higher. However, grade 3–4 cytopenia, including neutropenia, occurred in most patients and took several months to resolve. Moreover, infections occurred in 69% of patients, with approximately one-quarter of those being grade 3–4. Cilta-cel is also a second-generation CAR T cell product with a 4–1BB costimulatory domain but uses two variable binding regions to target two epitopes of BCMA. Cilta-cel was approved for the treatment of RRMM on the basis of the data of the pivotal CARTITUDE phase 1b/2 trial. The mPFS in CARTITUDE-1 was reported as an impressive 34.9 months (Lin et al. 2023). Median overall survival has still not been reached, with an estimated survival rate of 62.9% at 36 months. The outstanding length of cilta-cel’s PFS represents the longest observed compared with any previously reported therapy in patients with RRMM. However, this therapy shares a safety profile similar to that of ide-cel, with grade 3–4 cytopenia and infections commonly occurring (Berdeja et al. 2021, Martin et al. 2023, Munshi et al. 2023).

#### 5.1.2. Drawbacks to current therapy and aspects that need to be addressed for next-generation therapy.

Current CAR T cell therapy has undeniably provided patients with MM with exceptional options that also increase quality of life. However, toxicity (CRS requiring hospitalization and cytopenias), lack of activity in certain patient populations (extramedullary disease, high-risk subgroups), lack of persistence of CAR T cells, tumor relapse due to emergence of antigen-negative subclones, and vein-to-vein time are the most significant drawbacks. CAR T cell therapy requires manufacturing of an apheresis product, which currently creates a vulnerable window of time between previous treatment failure and instigation of CAR T cell therapy. In fact, in most trials to date, 10–20% of patients who underwent apheresis could not proceed to receive their product due to disease progression (Munshi et al. 2021, San-Miguel et al. 2023).

#### 5.1.3. Next-generation strategies to improve anti-MM CAR T cell therapy.

Several strategies are being evaluated to improve the clinical efficacy of CAR T cell therapy for MM: (*a*) CAR construct design, (*b*) cellular aspects of CAR product and the manufacturing process, (*c*) optimizing patient management during manufacturing time, (*d*) sequencing treatment with CAR T cells within a patient’s overall drug sequence, and (*e*) combining CAR T cell therapy with other anti-MM therapies.

##### 5.1.3.1. Construct design.

Many exciting molecular strategies are being pursued to improve CAR T cell construct design. While BCMA is still the most common target of anti-MM CAR T cell therapy, many new therapies target non-BCMA antigens. This avenue of development addresses the limitation of tumor antigen heterogeneity among tumor cells and the epitope loss or downregulation of antigen after exposure. Simultaneously targeting these antigens could also overcome this barrier. Non-BCMA antigens that are currently in clinical development (phase 1) target GPRC5D, SLAMF7, CD19, CD138, kappa light chain, CD38, CD44v6, and Lewis Y antigens. MCARH109 and BMS-986393, GPRC5D-targeting CAR T cell therapies, are being evaluated clinically and the early phase clinical results confirmed this as a promising target in MM (Bal et al. 2022; Mailankody et al. 2021a, 2022). These therapies have high response rates even in patient populations who have relapsed from previous BCMA-targeted immunotherapy. Dysgeusia, or taste disorder, or nail disorders are unique adverse events that occur with this therapy but seem manageable. Mitigating antigen loss might be necessary for durable CAR T cell therapy. Therefore, the current effort to combine binders as dual-targeting CARs (multispecific on the same CAR), or tandem-expressing CARs within the same cell, is especially promising. Several trials are evaluating this possibility, including GC012F, a dual-binding BCMA/CD19 CAR T cell therapy manufactured over 24–26 h on the FASTCAR platform (Du et al. 2022, Fu et al. 2023).

Additionally, while currently approved second-generation CAR T cells encode for only one T cell costimulatory domain (4–1BB), third-generation constructs contain two. Also in development are fourth-generation CAR constructs, commonly known as armored CARs or TRUCKs, that incorporate genes for cytokines, antibodies, or receptors (Alabanza et al. 2022, Duan et al. 2021). Finally, many developers are also incorporating human-derived and fully humanized CARs designed to reduce the risk of graft-versus-host disease (GvHD) and to prolong the persistence of the T cells. One of the first examples tested clinically, FHVH33, encodes for a fully human heavy-chain-only anti-BCMA binder to avert host recognition and shows deep and durable responses (Lam et al. 2020).

##### 5.1.3.2. Cellular properties of CAR products and improving the manufacturing process.

There are endless ways to improve the cellular properties of CAR T cells: generation of allogeneic CAR (allo-CAR) immune cells, optimization of ex vivo culture conditions to rapidly create T cell products, gene editing approaches to ablate inhibitory receptors or reduce fratricide, and the use of alternate effector cell types such as natural killer (NK) cells.

##### 5.1.3.3. Allo-CAR.

Allo-CAR T cell therapy has several attractive qualities for treating MM. These therapies are off the shelf and as T cells can be sourced from healthy individuals; they would lack the dysfunctional properties of those from patients with MM. However, the significant drawback of allogeneic CAR T cells is that they can cause GvHD and have the potential of being eliminated by the patient’s immune system as nonself. The UNIVERSAL phase I trial (Mailankody et al. 2021b) is investigating ALLO-715, an allogenic BCMA-directed CAR T cell construct, used in combination with TALEN technology to disrupt the T cell receptor to prevent GvHD and to disrupt the *CD52* gene, and in combination with ALLO-647, an anti-CD52, for lymphodepletion. The allogenic construct UCARTCS1, directed against SLAMF7, is also currently undergoing investigation in the clinical trial MELANI-01 (Mathur et al.2017). The clinical data for these trials are not mature, but results from similar trials in RR large B cell lymphoma or follicular lymphoma suggest a tolerable safety profile (Neelapu et al. 2020).

##### 5.1.3.4. Shortening CAR T cell manufacturing time.

Efforts to shorten the manufacturing time of CAR T cell products would clearly benefit patients by reducing the bridging time between therapies. These new protocols result in less-differentiated T cell phenotypes that are more enriched for naive and memory T cell subtypes than for terminally differentiated and effector T cells. CAR T cells created in these protocols have higher proliferative potential and therefore can be delivered at significantly lower doses in patients.

There are several ongoing studies of rapid manufacturing. BMS-986354 CAR T cells express a fully human BCMA-targeting CAR, derived from the retired Bristol Myers Squibb (BMS) or-vacabtagene autoleucel (orva-cel), and are manufactured rapidly using the proprietary NEX-T process, which takes 5–6 days. Early clinical results of patients with RRMM treated with a 10-fold-lower dose of CAR T cell than existing CAR T cell protocols showed a high rate of deep response [95% overall response rate (ORR), with 39% achieving a complete response (CR)], manageable toxicities, and achievement of similar pharmacokinetics despite the lower number of cells infused (Costa et al. 2022).

FasTCAR-T cells (GC012F, developed by Gracell Biotechnologies) express dual-targeting BCMA/CD19 CAR and are manufactured within 1 to 2 days. The simultaneous targeting of different antigens could also help overcome heterogeneous antigen expression or antigen loss. In the RRMM setting, FasTCAR-T cells have a safety profile similar to and a slightly stronger efficacy profile (93% ORR, with 82% achieving CR, and an mPFS of 38 months) than the BMS NEX-T process. In the transplant-eligible, newly diagnosed high-risk MM setting, patients treated with FasTCAR-T show 100% ORR with no grade 3 CRS or immune effector cell-associated neurotoxicity syndrome (ICANS) events (Derman et al. 2023, Du et al. 2022, Zhang et al. 2020). Finally, T-Charge (PHE885, developed by Novartis) CAR T cells express a BCMA-targeting CAR and are manufactured within approximately 2 days (Derman et al. 2023, Sperling et al. 2023). In the RRMM setting tested in phase I, patients receiving T-Charge CAR T cell therapy displayed 98% ORR, with 42% achieving CR. A small subset of patients had grade 3 CRS or ICANS, and dose-limiting toxicities were observed in 22% of patients.

##### 5.1.3.5. CAR–NK cells.

There is a growing interest in testing alternative effector cell types for redirection with a CAR. Of particular interest are NK cells, which are granulocytic innate lymphocytes that act in an antigen-independent manner to directly kill infected or malignant cells and stimulate other arms of the immune system through cytokine production (Biederstädt & Rezvani 2021, Khan et al. 2019). NK cell function is regulated by the target cell’s expression of activating or inhibiting ligands, including the presence of bound antibodies or downregulation of MHC class I molecules. Current MM therapies such as the mAbs daratumumab, isatuximab, and elotuzumab work in part by activating endogenous NK cell activities (and antibody-dependent cellular toxicity).

Because allogeneic NK cells do not cause GvHD, current NK cell therapy programs can mostly rely on allogeneic sources, avoiding the potential dysfunction of endogenous patients’ NK cells while reducing the time to vein. NK cells for clinical use and CAR engineering can be sourced from peripheral blood mononuclear cells, cord blood, immortalized cell lines, and induced pluripotent stem cells. Currently, immunotherapy programs from the MD Anderson Cancer Center and Takeda using cord blood NK cells and Fate Therapeutics using induced pluripotent stem cells are the most advanced in showing clinical efficacy. Of these, Fate Therapeutics has begun phase I clinical testing for patients with RRMM using their allogeneic NK cells, derived from a clonal, CD38 knockout, human-induced pluripotent stem cell line that expresses anti-BCMA or anti-CD38/SLAMF7 CAR; high-affinity, noncleavable CD16 (hnCD16); and IL-15/IL-15 receptor fusion protein (IL-15RF). They are testing this as a single agent or in combination with daratumumab (NCT05182073, NCT04614636).

As NK cells are short lived, they should be combined with other therapies to deepen or prolong responses; or with immunogenic therapies capable of promoting endogenous antitumor responses and epitope spreading, like in a post–autologous stem cell transplant, CAR T cell maintenance, checkpoint inhibitors; or concurrently with early doses of bispecific T cell engagers (TCEs).

#### 5.1.4. Sequencing treatment with CAR T cells.

An important question being actively addressed clinically is whether CAR T cell therapies should be initiated in earlier lines of treatment. Results of KarMMa-3 show that ide-cel therapy significantly prolonged mPFS and improved response compared with standard regimens in patients with triple-class-exposed RRMM who had received two to four prior regimens. However, the 13-month mPFS was disappointing in this less heavily pretreated patient population (Rodriguez-Otero et al. 2023). More impressive were the results from the CARTITUDE-4 study, in which patients with lenalidomide refractory MM (one to three lines of previous therapy) show a CR or better of 73.1% (in patients treated with cilta-cel) versus 21.8% (in patients treated with standard care) and have a significantly lower risk of disease progression or death (hazard ratio of 0.26) with cilta-cel than with standard care (San-Miguel et al. 2023).

#### 5.1.5. Combining CAR T cell therapy with other anti-MM therapies.

Combining CAR T cell therapy with standard of care (SoC) agents could be the most effective way to decrease tumor burden, increase CR, increase CR duration, and reduce relapse due to antigen loss. Furthermore, rational choices of SoC agents may also benefit the cellular CAR T cell function intrinsically. For example, by boosting IL-2 production, Ikaros degraders enhance CAR T cell activation and proliferation (Wang et al. 2018, Works et al. 2019). Ongoing clinical trials are combining CAR T cells with lenalidomide, lenalidomide maintenance, or gamma-secretase inhibitors to stabilize BCMA on the surface of tumor cells. A phase I trial using the gamma-secretase inhibitor crenigacestat with BCMA CAR T cells showed that it was well tolerated and significantly increased BCMA density on patients’ tumor cells (Cowan et al. 2023). A greater understanding of how combination with SoC agents and gamma-secretase inhibitors affects the safety, efficacy, and biology of the CAR T cell itself is needed.

### 5.2. Cell Engagers and Bispecific Antibodies

BsAbs are an off-the-shelf cell engager antibody-like drug designed to juxtapose cell types, leading to effector cell activation. Those far along in development for MM specifically engage T cells and recognize specific antigens on the surface of tumor cells, resulting in T cell activation, immunological synapse formation, and target cell apoptosis (Figure 3). The quality of the antitumor-redirected T cell response is determined in part by the affinity of the T cell/CD3 binding domain, the affinity of the tumor-targeting domain, target shedding and immunogenicity, the fitness of the patient’s native T cell repertoire, and the permissibility of the tumor microenvironment.

#### 5.2.1. Current T cell engager therapy.

Bispecific TCEs in clinical development in RRMM target the tumor antigens BCMA, GPRC5D, and FcRH5. Currently, teclistamab and talquetamab, both developed by Janssen, and elranatamab, developed by Pfizer, are the BsAbs approved by the FDA to treat MM (Lesokhin et al. 2023, Moreau et al. 2022), but many others are in clinical development. For teclistamab, the MajesTEC-1 study showed an ORR of 63% and a CR of 39.4%, with an mPFS of 11.3 months in patients with RRMM. While CRS was the most common safety concern, less than 1% of patients had CRS of grade 3 or higher. Conversely, grade 3–4 cytopenia, including neutropenia, occurred in most patients and took several months to resolve. Also, infections occurred in 76% of patients, with approximately 45% of those being grade 3–4. Other BCMA × CD3 BsAbs in late clinical development for RRMM have strong efficacy and safety profiles similar to those of teclistamab: alnuctamab (BMS) (Costa et al. 2019, Seckinger et al. 2017), linvoseltamab (Regeneron) (DiLillo et al. 2021, Zonder et al. 2022), and ABBV-383 (AbbVie) (D’Souza et al. 2022). Of note, alnuctamab is designed as an asymmetric 2 + 1 binder with two binding sites for BCMA, which may help mitigate epitope loss as a resistance mechanism. Taken together, these therapies provide a vital off-the-shelf immunotherapy to treat patients with relapsed disease, and more research is justified to expand the therapeutic options and increase durable remissions.

#### 5.2.2. Drawbacks to current therapy and aspects that need to be addressed for next-generation therapy.

Collectively, the limitations of bispecific TCEs include primary resistance (especially in patients with high-risk disease or extramedullary disease), lack of durable remissions, extended cytopenia, and increased rates of opportunistic infections, the last of which is almost double that seen with CAR T cells and likely due to the continual activation experienced by the entire T cell repertoire.

Bispecific TCEs rely on activation of the native T cell repertoire to induce a polyclonal antitumor response. Therefore, they are dependent on the patient’s T cell quality and function, which show high interpatient variability and are impaired in the setting of MM and prior lines of therapy (Cohen et al. 2020). The antitumor response elicited from TCEs is also presumably affected by the degree of immunosuppression within the tumor microenvironment. Understanding these mechanistic correlates of response is currently an unmet need for improving responses and durable remission to TCEs.

Research into the determinants of primary and acquired resistance to bispecific TCEs is vital to guide next-generation development. So far, these data justify predictive immune monitoring and strategies to condition the immune repertoire to improve future, more durable TCE therapy. Preclinical and preliminary clinical trial correlate research suggests that high tumor burden and the baseline characteristics of patients’ T cells correlate with response and primary resistance to TCEs (Cortes-Selva et al. 2022, Friedrich et al. 2023, Girgis et al. 2023, Meermeier et al. 2021). Regarding T cell characteristics, primary resistance is associated with an unfavorable immune profile, including the abundance of exhausted-like CD8+ T cell clones, higher frequency of regulatory T cells, and lower frequency of naive T cells. A longer mPFS in the MajesTEC-1 trial was associated with fewer PD1+CD8+ and regulatory T cells peripherally, and also with a lower frequency of activated CD4+ T cells in the bone marrow. Taken together, these are the first proposed predictive markers of response to TCE therapy. Recent data suggest that acquired resistance to TCEs involves the accumulation of exhausted T cells, tumor intrinsic adaptations such as loss of target epitope through structural and point mutations, and downregulation/loss of MHC class I molecules (Friedrich et al. 2023, Lee et al. 2023). Of interest, mutations in the extracellular domain of BCMA do not affect its pro-survival signaling but variably alter the binding of TCEs (Lee et al. 2023). To address these limitations, researchers are pursuing several strategies.

##### 5.2.2.1. Design and constructs.

Multiple other tumor targets for TCEs, including GPRC5D, FcRH5, and CD38, are being pursued preclinically and clinically. Talquetamab, recently approved by the FDA for treating RRMM, is a GPRC5D × CD3 TCE that displays an ORR of 73% and a CR in 32% of patients with an mPFS of 1 year (Chari et al. 2022b). The rates of grade 3 or higher CRS or ICANS remain below 2% of treated patients. Preliminary results with cevostamab, an FcRH5 × CD3 TCE, show an ORR of 56.7%, with a CR in 8% of patients, and a safety profile in line with that of other TCEs (Lesokhin et al. 2022).

One strategy to increase the durability of responses to TCEs is to increase the agent’s half-life and decrease its size. A phase I study of HPN217 evaluates a half-life-extended TCE developed by Harpoon Therapeutics (Abdallah et al. 2022). In addition to BCMA × CD3, this TCE binds albumin, allowing for a long half-life without an Fc portion. While data are not mature enough to answer whether this TCE provides more durable anti-MM responses, so far it displays deep response rates and a manageable safety profile.

A strategy to mitigate tumor escape through antigen loss is the development of a multispecific TCE that binds multiple tumor targets. One promising example under preclinical development by Ichnos Sciences is ISB-2001, a trispecific TCE that engages BCMA × CD38 × CD3 (Pihlgren et al. 2022). By having the ability to bind BCMA and CD38 simultaneously, TCE’s avidity to bind to the target tumor is advantageous and was associated with low on-target, off-tumor activity compared with the combination of the CD38-specific and BCMA-specific TCEs constructed with the same binding domains.

##### 5.2.2.2. Dosing and frequency of administration.

Further studies are required to determine the optimal dosing and scheduling strategies to support the persistence of the favorable T cell profile observed in good responders while avoiding the evolution of the T cell exhaustion and antigen escape that underpin acquired resistance. Recent data report a higher incidence of antigen loss/mutations in patients with MM receiving TCEs than in those receiving CAR T cells, suggesting that clonal evolution is more likely to occur under continual therapeutic pressure than with a single administration of CAR T cells (Lee et al. 2023). Spreading out dosing may minimize hematological and infectious toxicities by allowing the T cell compartment to return to homeostasis instead of being chronically activated. The discontinuation of TCE therapy, especially in the setting of persistent minimal residual disease-negative response, might enable the recovery of immune cell subsets with anti-MM activity while avoiding selection of antigen-mutated MM clones. However, there is currently an unmet need to learn the type of dosing required to achieve this or to adjust TCE dosing on the basis of individualized patient responses.

#### 5.2.3. Combinations.

To further optimize bispecific TCE treatment, several new strategies are under investigation. Impressive preclinical studies suggest that combination with cytotoxic chemotherapy can overcome a high MM tumor burden that negatively affects the efficacy of TCEs and can lead to T cell exhaustion, while also boosting endogenous T cell immunity (Meermeier et al. 2021). Combinations with daratumumab with a suggested mechanism of action of limiting the activity of regulatory T cells (Krejcik et al. 2016, Rodríguez-Otero et al. 2022) are being supported by Janssen, Regeneron, and Pfizer (Grosicki et al. 2022). The combination of pavurutamab (AMG 701) with pomalidomide and dexamethasone is being evaluated in patients with RRMM after three or more lines of prior treatments (Harrison et al. 2020). Regeneron is supporting a new phase I clinical trial that combines its anti-BCMA/CD3 TCE with nine other drugs (Rodríguez Otero et al. 2022). As noted in Section 5.1.5, gamma-secretase inhibitors increase membrane BCMA expression and can increase the efficacy of TCEs in vitro. However, preliminary data of nirogacestat in combination with teclistamab in MajesTEC-2 suggest excessive therapeutic toxicity (Offner et al. 2023).

### 5.3. Targeting the Microenvironment

An emerging theme of recent research that is incorporating high-dimensional single-cell and spatial technologies into MM samples is the interpatient variability of the degree of dysfunction in immune microenvironment cell composition and cell states. The dysfunction particularly involves accumulation of terminally differentiated, exhausted T cells and subsequent depletion of those expressing naive and stem-like markers (Dhodapkar 2023, Foster et al. 2022, Zavidij et al. 2020). Many changes in patients with MGUS and those with newly diagnosed MM are already noted (Bailur et al. 2019, Liu et al. 2021, Zavidij et al. 2020). The immune repertoire is shaped by MM tumor load, previous therapy, and disease stage (Bailur et al. 2019, Ledergor et al. 2018, Tirier et al. 2021). While this dysfunction certainly has implications for patient infection prevention, such as in the context of SARS-CoV-2 vaccination (Azeem et al. 2023), we speculate that this should also guide rational designs of more durable next-generation immunotherapy. As multiple T cell–based therapies enter earlier lines of therapy, it may be that differences in T cell states at baseline or quality of endogenous adaptive immune responses will translate into differences in outcomes for T cell redirection with BsAbs, CAR T cell therapy (Dhodapkar 2023), and next-generation immunotherapy.

#### 5.3.1. Checkpoint inhibitors.

Checkpoint inhibitors are a revolutionary pillar of modern cancer therapy used to treat many types of malignancies, especially through the PD1/PD-L1 or CTLA-4 axes. Immune checkpoint blockade reduces immune dysregulation in the tumor microenvironment and reactivates endogenous adaptive immunity. However, the use of checkpoint inhibitors in MM has not shown clinical benefit so far. In clinical trials (KEYNOTE trials 183 and 185) (Mateos et al. 2019, Usmani et al. 2019), the combination of Ikaros degraders with pembrolizumab resulted in increased mortality without signs of efficacy, which halted the trials. While the reason underpinning these safety concerns remains unsolved, it warrants caution for future checkpoint blockade with Ikaros degrader combination clinical trials in MM. TIGIT is an inhibitory checkpoint receptor expressed on subsets of T cells and NK cells. In MM, TIGIT expression increases as the disease progresses, correlates with defective T cell effector functions, and is more highly expressed in bone marrow T cells of mice and humans than other immune checkpoints are (Guillerey et al. 2015, 2018; Kurtulus et al. 2019; Minnie et al. 2018). We conjecture that TIGIT blockade may be a safer therapeutic due to preclinical studies showing that *Tigit* knockout mice do not have appreciably compromised immune homeostasis, in contrast to *Pdcd1* or *Ctla4* knockout mice. Of note, the MM Research Consortium is sponsoring a clinical trial in an RRMM setting to evaluate anti-TIGIT (or anti-LAG3) safety and efficacy as single agents or in combination with pomalidomide plus dexamethasone (MyCheckpoint, NCT04150965). More research is warranted to define the potential therapeutic opportunities for checkpoint inhibitors in MM, including which checkpoints are most relevant for the MM niche, whether checkpoint blockade may combine well with SoC agents that stimulate immunogenic cell death, or whether checkpoint blockade should be incorporated into novel CAR constructs or bispecific antibody designs.

#### 5.3.2. Promoting phagocytosis.

The signal regulatory protein α (SIRPα)–CD47 axis is an innate immune checkpoint that enables cancer cell escape from macrophage phagocytosis (Jiang et al. 2021). SIRPα expression is limited to macrophages, dendritic cells, and neutrophils, which are enriched in the tumor microenvironment. CD47 expression on tumors binds to SIRPα, which leads to an antiphagocytosis signal. Many CD47-blocking antibodies are designed to also provide a prophagocytosis signal by activating Fc receptors. This axis has not yet been targeted successfully in clinical trials for MM, but several companies are currently pursuing phase I/II trials. AO-176 is a humanized mAb against CD47 in phase I/II investigation as a single agent or in combination with dexamethasone or bortezomib (Arch Oncology) (Andrejeva et al. 2021). Maplirpacept, or PF-07901801, is a SIRPα–Fc fusion biologic designed to block CD47 while delivering an “eat me” signal to macrophages. It is currently under phase I/II clinical investigation as a single agent or in combination with SoC agents, such as daratumumab (NCT03530683, NCT05139225), in patients with MM.

#### 5.3.3. Multiple actions of IAP antagonists.

Antagonists of the inhibitor of apoptosis proteins (IAPs) were developed to suppress their function that promotes mitochondrial apoptosis pathway in tumors. They function as a molecular glue to redirect the ubiquitin ligase activity of cIAP1 and cIAP2 to cause their auto-ubiquitination and subsequent degradation. As these proteins are negative regulators of NFκB inducing kinase (NIK), their degradation results in constitutive activation of the alternative NFκB pathway. Due to cellular IAPs’ simultaneous role in regulating NFκB signaling, treatment with IAP antagonists sensitizes tumor cells to apoptotic signals while increasing alternative NFκB signaling in T cells and the tumor microenvironment in promising ways that may support immunotherapy for MM (Chesi et al. 2016, Gu et al. 2021, Roehle et al. 2021). These agents are under preclinical development for the treatment of cancer, and several studies highlight their promise for treating the MM microenvironment to promote anti-MM immune responses. It is attractive to think of using one of these agents in combination with anti-MM CAR T cell therapy in which downregulation or loss of MHC class I molecules has been recently associated with acquired resistance.

## 6. CONCLUSIONS

Despite the early excitement noting unprecedented rates of CR following CAR T cells and TCEs in MM, we now realize that T cell–redirected therapy is still not curative and that patients with MM eventually relapse as they do after conventional therapy. In both cases, tumor heterogeneity is the culprit, providing a source of antigen-negative or mutated cells, while loss of T cell fitness impairs antitumor immune control. However, the many rational approaches to improve upon current therapeutic strategies together with the use of simultaneous combination therapy to different targets, applied in a low-tumor-burden setting, offer promise for the future. The lack of demonstrated curative potential suggests caution should be used when introducing these strategies in premalignant MGUS and SMM.
